# Genetic Effects of Chicken *Pre-miR-3528* SNP on Growth Performance, Meat Quality Traits, and Serum Enzyme Activities

**DOI:** 10.3390/ani15152300

**Published:** 2025-08-06

**Authors:** Jianzhou Shi, Jinbing Zhao, Bingxue Dong, Na Li, Lunguang Yao, Guirong Sun

**Affiliations:** 1 School of Life Science, Nanyang Normal University, Nanyang 473061, China; shijian1109@163.com (J.S.); 20122031@nynu.edu.cn (J.Z.); nanyangshiyuan2025@163.com (B.D.); lina330077@163.com (N.L.); lunguangyao@163.com (L.Y.); 2 The Shennong Laboratory, Zhengzhou 450046, China; 3 Henan Field Observation and Research Station of Headwork Wetland Ecosystem of the Central Route of South-to-North Water Diversion Project, Nanyang 473061, China; 4 Henan Provincial Engineering and Technology Center of Health Products for Livestock and Poultry, Nanyang 473061, China; 5 College of Animal Science and Technology, Henan Agricultural University, Zhengzhou 450002, China

**Keywords:** microRNAs (miRNAs), single-nucleotide polymorphism (SNP), F_2_ resource population, *pre-miRNA-3528*, growth performance, carcass traits, meat quality traits, serum enzyme activities

## Abstract

The aim was to investigate the genetic effects of a SNP located in the precursor region of *gga-miR-3528*. (1) Single-nucleotide polymorphisms within precursor regions of microRNAs play crucial biological roles. (2) Utilizing a Gushi–Anka F_2_ resource population (*n* = 860), we screened and validated miRNA SNPs. A SNP mutation in the *miR-3528* precursor region was identified. Specific primers were designed to amplify the polymorphic fragment. Genotyping was performed for this individual SNP across the population, using the MassArray system. Association analyses were conducted between this SNP and chicken growth and body measurement traits, carcass traits, meat quality traits, and serum enzyme activities. (3) The rs14098602 (+12 bp A > G) was identified within the precursor region of *gga-miR-3528*. Significant associations (*p* < 0.05) were observed between this SNP and chicken growth traits (body weight at the age of 0 day, body weight at the age of 2 weeks, and body weight at the age of 4 weeks), carcass traits (evisceration weight), meat quality traits (subcutaneous fat rate and pectoral muscle density), and serum enzyme activities (total protein, albumin, globulin, cholinesterase, and lactate dehydrogenase). (4) These findings suggest that the polymorphism at rs14098602 may influence chicken growth, meat quality, and serum biochemical indices, through specific mechanisms. The *gga-miR-3528* gene likely plays an important role in chicken development. Therefore, this SNP can serve as a molecular marker for genetic breeding and auxiliary selection of growth-related traits, facilitating the rapid establishment of elite chicken populations with superior genetic resources.

## Simple Summary

[Background] Single-nucleotide polymorphisms (SNPs) within microRNA (miRNA) precursor regions exert critical biological functions. This study elucidated the genetic effects of a SNP located in the precursor region of *gga-miR-3528*. [Method] Utilizing an F_2_ resource population derived from Gushi–Anka chickens (*n* = 860), SNPs within miRNA precursors were systematically screened. [Result] A specific SNP locus rs14098602 (+12 bp, A > G) was identified in the precursor region of *miR-3528*. This polymorphism was significantly associated with multiple indicators such as the growth performance, carcass traits, meat quality traits, and serum enzyme activities of chickens (*p *< 0.05). These findings demonstrate significant associations between the rs14098602 polymorphism and key phenotypic traits (growth performance, meat quality attributes and serum enzyme activities), indicating its functional role in regulating chicken development. [Conclusions] Consequently, the *gga-miR-3528* gene appears to play an important role in chicken developmental processes. This SNP therefore represents a viable molecular marker for genetic breeding strategies and marker-assisted selection targeting growth-related traits, facilitating the efficient development of elite chicken populations with enhanced genetic merit.

## 1. Introduction

MicroRNAs (miRNAs) are short non-coding RNAs that constitute a crucial component of the non-coding RNA repertoire. They play pivotal roles in the post-transcriptional regulation of gene expression. This regulatory function is achieved through binding to the 3′ untranslated regions (3′ UTRs) of target genes, enabling miRNAs to exert critical influences on fundamental physiological processes such as cellular growth, differentiation, and metabolism [1,2]. Recent studies have increasingly demonstrated that miRNA biogenesis and function are modulated by Single-Nucleotide Polymorphisms (SNPs). As the most prevalent type of genetic variation, SNPs are ubiquitously distributed across genomic regions, including miRNA genes themselves and the regulatory sequences of their target genes [3,4]. This discovery provides a novel perspective for understanding the manifestation of genetic traits.

Single-Nucleotide Polymorphisms (SNPs), as a significant category of genetic variation [5], constitute a crucial aspect in the study of genetic diversity in livestock and poultry. SNPs can reveal genetic differences among distinct breeds, holding important implications for understanding the evolutionary history and genetic background of these species. Consequently, SNP research has gained widespread attention in recent years within the field of livestock and poultry genetics and breeding. SNP loci are closely associated with key economic traits such as growth traits [6], reproduction traits [7], and meat quality traits [8] in livestock and poultry, directly influencing their economic value. As an important molecular marker [9], the application of SNPs continues to expand in animal breeding and meat quality improvement. Therefore, analyzing SNPs to uncover the genetic factors influencing growth and development traits holds significant practical importance. Developing genetic markers for livestock and poultry breeding serves as a tool for molecular breeding, which can accelerate breeding progress and provide a theoretical foundation for breeding programs.

A resource population is a segregating population generated through specific mating designs. This population must possess complete pedigree records and phenotypic records of traits. Crucially, the major traits of interest should exhibit segregation and recombination within this population. Resource populations are primarily employed for genetic map construction and functional gene localization [10,11,12].

This study utilized the Gushi × Anka F_2_ resource population as the research subject to identify SNPs in *miRNA-3528* and conduct association analyses with multiple phenotypic datasets, including growth and development traits, meat quality traits, and serum biochemical parameters. The objective was to establish the identified miR-SNPs as potential molecular targets for improving economic traits and meat quality. This work aims to provide a significant theoretical foundation for chicken genetic breeding and offer valuable insights for future genetic improvement strategies.

## 2. Materials and Methods

### 2.1. Establishment of the Chicken Resource Population

The study utilized an established Gushi × Anka F_2_ chicken resource population (*n* = 860), originally developed through a published experimental design. The details have been previously described by Han et al. [13,14,15]. Specifically, the F_2_ resource population was generated by crossing slow-growing Chinese indigenous Gushi chickens (selected for superior meat traits) with fast-growing Anka broilers. The F_2_ resource population was established through a half-sibling distant-cross design between Gushi and Anka chickens, comprising seven families (designated A, B, C, D, E, F and G): 4 directional crosses (Anka♂ × Gushi♀) and 3 reciprocal crosses (Gushi♂ × Anka♀). F0 founders were selected from purebred stocks of dual-purpose (egg-laying and meat-producing) Gushi chickens and meat-type Anka chickens, based on breed-standard phenotypes, high egg production, moderate body weight, and confirmed purebred status. Crosses were conducted at a 1♂: 6♀ ratio to ensure heterozygosity across all loci in F_1_ progeny. The F_2_ generation was generated by mating single roosters selected from each F_1_ family with hens derived from non-sibling families at a 1♂: 9♀ ratio. As discussed above, this F_2_ resource population was generated by crossing 63 F_1_ hens and 7 F_1_ cockerels. The F_2_ chicken resource population (*n* = 860) was housed in cages under standardized conditions with ad libitum access to feed and water until 12 weeks of age. All the experimental chickens were humanely euthanized at 84 days post-hatch. All animal experiments were approved by the Institutional Animal Care and Use Committee of Protocol, and conducted in strict accordance with the approved guidelines.

### 2.2. Measured Traits

Body weight (BW): chickens were individually weighed and recorded every two weeks starting at hatch (0 weeks) and continuing through 2, 4, 6, 8, 10, and 12 weeks (slaughter age).

Body size traits: at designated growth stages, measurements included shank length (SL), shank girth (SG), chest depth (CD), chest breadth (CB), breast-bone length (BBL), pectoral angle (PA), body slanting length (BSL), and pelvis breadth (PB).

Carcass traits: following slaughter, the following traits were accurately determined: semi-evisceration weight (SEW—carcass weight excluding reproductive organs, spleen, pancreas, gallbladder, crop, esophagus, intestine, and trachea), evisceration weight (EW—SEW excluding abdominal fat, gizzard, liver, head, proventriculus, feet, and heart), breast muscle weight (BMW), leg muscle weight (LMW), and carcass weight (CW). The measurement methods have been previously described by Han et al. [13].

### 2.3. Serum Biochemical Indicators

Blood samples (5 mL) were collected for all 860 F_2_ chickens from the jugular vein during slaughter at 12 weeks of age and transferred into centrifuge tubes. The tubes were tilted at an angle at room temperature for 30 min to allow serum separation. Subsequently, samples were centrifuged at 3000× *g* for 30 min. The isolated serum was stored at −80 °C until analysis of serum biochemical indicators.

There are a total of 17 biochemical indicators, including alanine aminotransferase (ALT), aspartate aminotransferase (AST), γ-glutamyl transpeptidase (γ-GT), alkaline phosphatase (AKP), cholinesterase (CHE), creatine phosphokinase (CPK), lactate dehydrogenase (LDH), amylase (AMY), total protein (TP), albumin (ALB), globulin (GLOB), creatinine (CRE), glucose (GLU), total cholesterol (TC), triglycerides (TG), high-density lipoprotein (HDL), and low-density lipoprotein (LDL). The serum biochemical indicators’ activities were analyzed and determined with the kits purchased from Nanjing (Nanjing, China). The instructions in the kit manual were followed, to carry out the operation. The specific measuring methods were as previously detailed by Han et al. [16].

### 2.4. Polymorphism Detection

Using EDTA-anticoagulated venous blood, genomic DNA from 860 individuals of the Gushi × Anka F_2_ chicken reference family was extracted with phenol–chloroform and a commercial DNA kit (Tiangen Biotech, Beijing, China). Prior to MassArray matrix-assisted laser desorption/ionization-time of flight (MALDI-TOF) mass genotyping [17], DNA concentrations were accurately measured according to platform specifications. All aliquots were archived at −80 °C for future studies. A randomly selected subset of 100 F_2_ chicken genomic DNA samples was pooled at equal volumes, after standardization to uniform working concentrations. The genomic sequence of chicken *pre-miR-3528* (GenBank: MI0015379), located on chromosome 17, was retrieved from miRBase release 20.0. Commercial sequencing of the F_2_ chicken resource population pooled DNA was performed by Sangon Biotech (Shanghai, China) for genome-wide polymorphism screening. PCR primers (Forward: 5′-CAGTGTTGTGTACGTTGTCTGCTC-3′; Reverse: 5′-GTCAAGAAGTTGCTGACAGCATTG-3′) targeting the *pre-miR-3528* precursor region were designed based on genomic coordinates NC_052548.1: 8329802..8329898 for SNP detection. The length of the target fragment is 412 bp. These primers amplified the target genomic region encompassing *pre-miR-3528*. The reaction system for PCR amplification is 25 μL: 2 × Taq PCR Master Mix (Sangon Biotech, Shanghai, China), 12 μL; forward primer (10 pmol/L), 1 μL; reverse primer (10 pmol/L), 1 μL; DNA template (100 ng/μL) 0.5 μL; ddH_2_O, 10.5 μL. The reaction procedure for PCR amplification is pre-denaturation at 94 °C for 5 min; denaturation at 94 °C for 30 s, annealing at 60 °C for 30 s, extension at 72 °C for 30 s, 30 cycles; extend at 72 °C for 10 min. Sequencing of PCR amplification products (Sangon Biotech, Shanghai, China).

### 2.5. Genotyping

DNA samples underwent MALDI-TOF MS-based SNP genotyping at a certified commercial facility. In the Gushi × Anka F_2_ chicken resource population, genotyping of the rs14098602 SNP (+12 bp A > G) located within *pre-miR-3528* was performed using the MassArray-iPLEX GOLD platform (Sequenom Inc., San Diego, CA, USA). SNP genotyping employed specific primer pairs for amplification and a single-base extension primer, both designed using Professional Assay Design software (version 3.1). The primer sequences used were as follows: upstream PCR amplification primer for the *pre-miR-3528* SNP (5′-ACGTTGGATGCATGGCACTACAGCCATATC-3′); downstream PCR amplification primer for the *pre-miR-3528* SNP (5′-ACGTTGGATGTGAGTCGCAGTGGATACAAG-3′); and single-base extension primer (5′-GCTCATGCATTACACAG-3′). The *pre-miR-3528* SNP was genotyped via MALDI-TOF MS, following the operation manual. Peak area and call rate data from the genotyping assay were collected. Allele designation was performed automatically by the manufacturer-provided Assay Design 3.1 software (Sequenom Inc., San Diego, CA, USA).

### 2.6. Secondary-Structure Prediction of Pre-miR-3528

The most stable secondary structure (minimum free energy) of *pre-miR-3528* harboring the rs14098602 SNP (A > G) in chickens was determined using the online M-fold web server (http://www.unafold.org/mfold/applications/rna-folding-form-v2.php, accessed on 3 August 2025). The magnitude of the free energy difference between the A and G alleles of *gga-miRNA-3528* (*Gallus gallus*) was used to assess their influence on the secondary structure of *pre-miR-3528*.

### 2.7. Statistical Analysis of the Data

SPSS 20.0 was used to analyze correlations between the *pre-miR-3528* SNP and growth traits, development traits, meat quality traits, and biochemical indicators in the Gushi × Anka F_2_ chicken resource population. Associations between gene polymorphisms and economic traits in the F_2_ chicken resource population were analyzed using a linear mixed model implemented in SPSS 20.0. Association analyses between the polymorphism and F_2_ population economic traits employed two linear mixed models. Model I assessed growth, development, meat quality, and biochemical traits, while Model II evaluated carcass traits with carcass weight as a covariate to correct for body weight effects. Significant genotype effects underwent Bonferroni-corrected multiple comparisons. The models were structured as follows:

Model I: Y_ijkl_ = μ + G_i_ + S_j_ + H_k_ + f_l_ + e_ijklm_

Model II: Y_ijkl_ = μ + G_i_ + S_j_ + H_k_ + f_l_ + b (W_ijklm_ − W) + e_ijklm_

Y_ijkl_: observed measured value; μ: overall mean; G_i_: fixed effect of genotype (i = 3 levels: AA, AG, GG); f_l_: random effect of the resource population (l = 7, 7 reference families), s_j_: fixed effect of sex (j = 2 levels); H_k_: fixed effect of hatch (k = 2 levels); b: the regression coefficient of carcass weight; W_ijklm_: the individual carcass weight; W: average carcass weight; e_ijklm_: random error term. Statistical significance was defined as *p *< 0.05 for fixed effects.

## 3. Results

### 3.1. Detection of Gga-miRNA-3528 Gene Polymorphism

**Figure 1:**
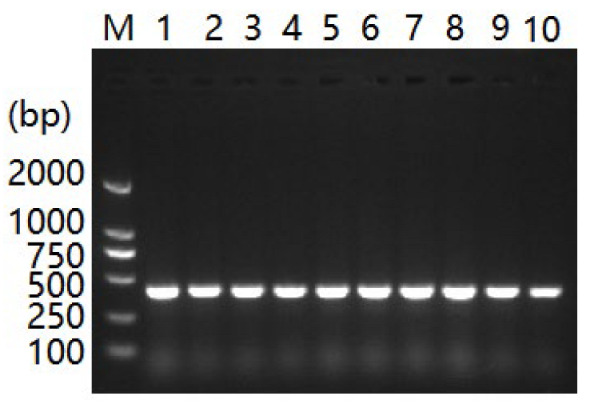
PCR products. M: marker; 1–10: lanes.

**Figure 2:**
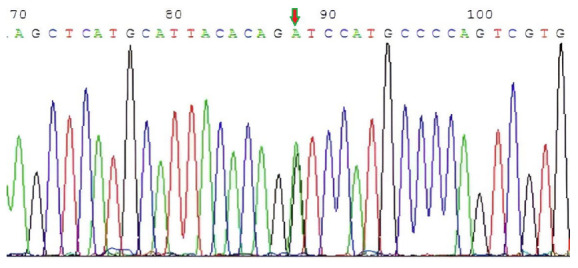
The partial DNA sequencing results of *miR-3528* precursor region. Arrow: SNP rs14098602 (A > G) (+88 bp). The area pointed to by the arrow represents the mutation site.

**Figure 3:**
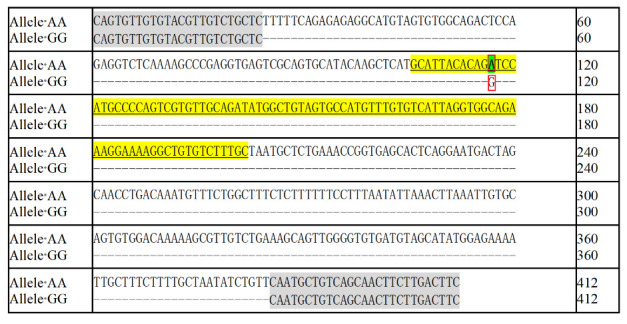
Sequence result of PCR products. The shaded parts represent primers, the yellow underlined parts represent *miRNA-3528* sequences, and the boxes represent mutants.

The 1.2% agarose gel electrophoresis pattern of the PCR amplification product (Figure 1) and the sequencing results of the purified and recovered target fragment were detected by the polymorphism of the *gga-miRNA-3528*. The PCR purification products were sequenced, and the sequencing peak maps were analyzed and sequence-aligned. The arrows on the sequencing map represent the mutation site A > G, which is located at +88 bp (Figure 2). There was an A/G polymorphism at position 12 of the *miRNA-3528* gene precursor. The results are shown in Figure 3. In conclusion, PCR-based sequencing confirmed an A > G mutation at +12 bp in *pre-miR-3528* within the F_2_ chicken resource population, validated against the published chicken genome reference.

### 3.2. Pre-miR-3528 SNP Genotyping

**Figure 4:**
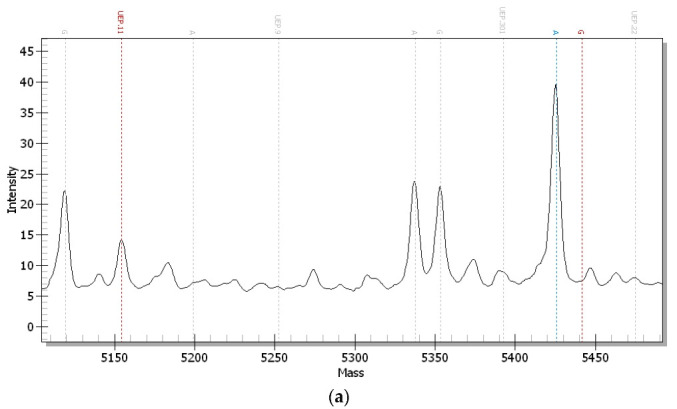

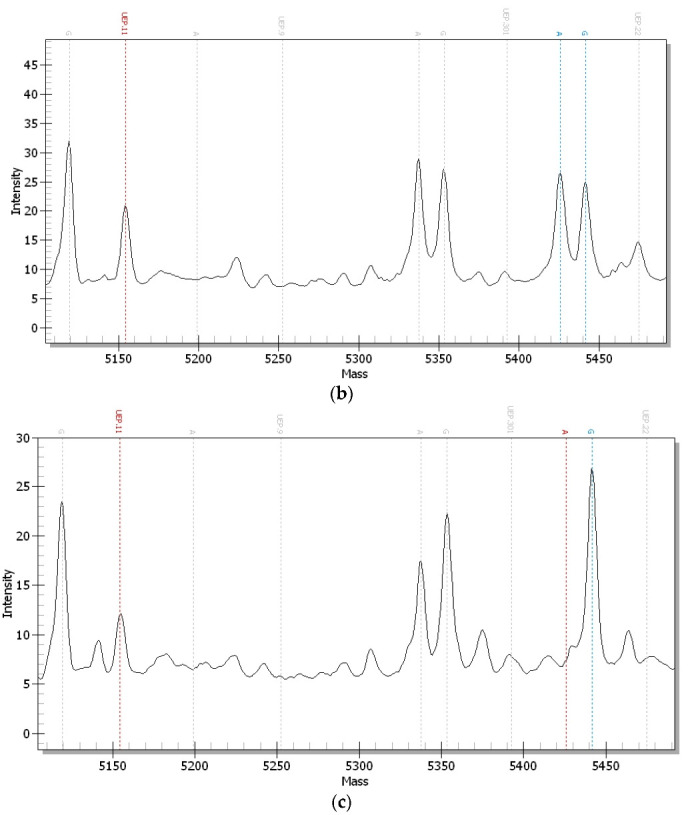
Characteristic mass spectra of different genotypes of *gga-miR-3258*. Structural prediction of *pre-miR-3528*. (**a**) AA genotype; (**b**) GG genotype; (**c**) AG genotype.

Figure 4 displays the mass spectra of three variant genotypes (AA, AG, andGG). Allele frequencies for the *pre-miR-3528* SNP in this F_2_ resource population were 0.8071 (A) and 0.1929 (G), while genotype frequencies reached 0.6484 (AA), 0.3175 (AG), and 0.0341 (GG).

### 3.3. Structural Prediction of Pre-miR-3528

**Figure 5:**
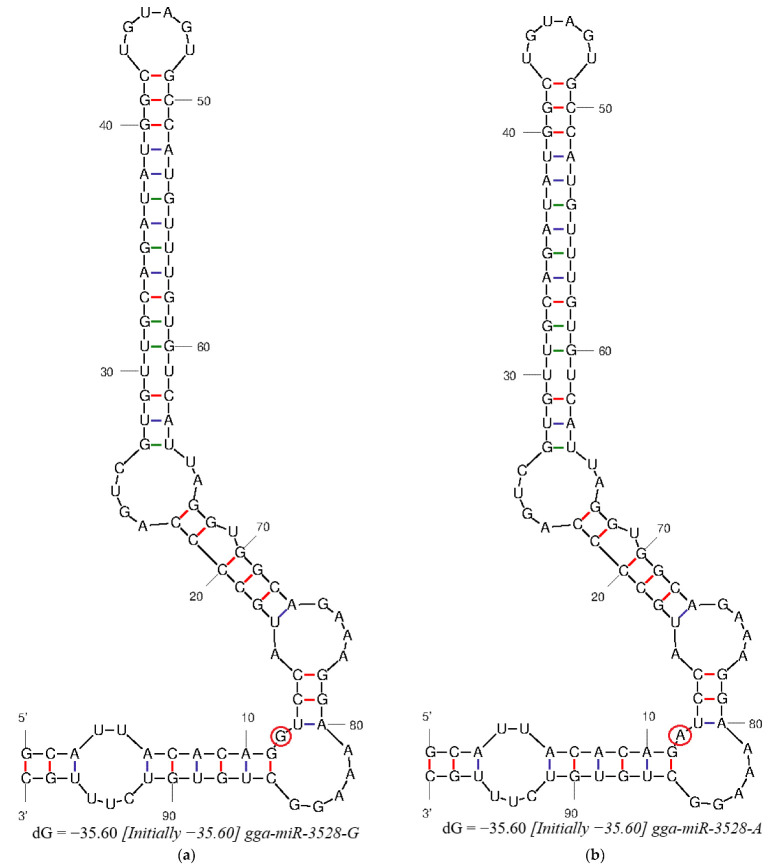
Prediction of the secondary structure of different alleles (G/A) of *miRNA-3528*. (**a**) Wild type; (**b**) mutant type. The area marked by the red circle represents the site of genetic mutation.

M-fold predicted secondary structural changes in *pre-miR-3528* RNA due to SNP allelic variation. Structural prediction revealed that the A > G SNP at position +12 bp in *pre-miR-3528* neither significantly altered its secondary structure nor changed the minimum free energy (ΔG = −35.6 kcal/mol), as shown in Figure 5. Rs14098602 (A/G) did not affect the stability of the secondary structure of *gga-miR-3528*.

### 3.4. Association Analysis of the Pre-miR-3528 SNP with Multiple Traits

Association analysis between different *gga-miR-3528* genotypes and carcass traits in the F_2_ resource population was performed, with the results presented in Table 1. The analysis revealed a significant association (*p* < 0.05) between the SNP in the *gga-miRNA-3528* gene and evisceration weight (EW) at 12 weeks of age. Furthermore, individuals with the GG genotype exhibited greater values for evisceration weight (EW), breast muscle weight (BMW), leg muscle weight (LMW), and carcass weight (CW) compared to those with the AA or AG genotypes, with GG > AG > AA.

Association analysis between different *gga-miR-3528* genotypes and growth traits in the F_2_ resource population was conducted, with results presented in Table 1. The analysis revealed a significant association between the SNP in the *gga-miRNA-3528* gene and body weight (BW). Specifically, individuals with the GG genotype exhibited significantly greater 0BW (hatch weight), body weight at 2 weeks (GG > AG > AA, *p* < 0.05), and body weight at 4 weeks (GG > AG and GG > AA, *p* < 0.05) compared to those with the AG or AA genotypes. Furthermore, the GG genotype consistently demonstrated higher body weight than both the AA and AG genotypes at subsequent stages, including 6, 8, 10, and 12 weeks of age.

Association analysis between the SNP (single-nucleotide polymorphism) in the *gga-miR-3528* gene and chicken body size traits was performed, with results summarized in Table 2. The analysis revealed that individuals with the GG genotype exhibited greater values for the following traits compared to those with the AG or AA genotypes: shank length (0SL, 4SL, 8SL, 12SL), shank girth (4SG, 8SG, 12SG), chest depth (8CD, 12CD), chest breadth (8CB), breast-bone length (4BBL), body slanting length (8BSL,12BSL), pelvis breadth (8PB, 12PB).

Association analysis between the SNP in the *gga-miR-3528* gene and meat quality traits in the F_2_ resource population was conducted, with results detailed in Table 3. The analysis revealed the following associations: subcutaneous fat rate (SFR): GG genotype exhibited a significant decrease (GG < AG < AA, *p *< 0.05). Pectoral muscle density (PMD) and pectoral muscle area percentage (PMAP): GG genotype showed a significant increase (GG > AG > AA, *p *< 0.05). Leg muscle area percentage (LMAP) and weight after de-feathering (WAD): GG genotype demonstrated higher values (GG > AG > AA). Intermuscular fat width (IFW) and subcutaneous fat thickness (SFT): values followed the pattern AG < GG < AA. Pectoral muscle pH (PMpH) and leg muscle pH (LMpH): GG genotype yielded the lowest values (GG < AG < AA). Leg muscle density (LMD) (GG > AA) and pancreas weight ratio (PWR) (GG > AA, GG > AG): GG genotype displayed higher values than the AA genotype and AG genotype.

Association analysis between the *gga-miR-3528* SNP and serum enzyme activities in chickens was performed, with results presented in Table 4. The analysis revealed the following associations: total protein (TP) and cholinesterase (CHE) exhibited highly significant differences among genotypes (*p *< 0.01). Albumin (ALB), globulin (GLOB), and lactate dehydrogenase (LDH) exhibited significant differences among genotypes (*p *< 0.05). ALB, GLOB, and CHE: the GG genotype exhibited significantly lower levels than AG and AA genotypes, with GG < AG < AA. TP: the GG genotype showed significantly lower levels than both AG and AA genotypes (GG < AG, GG < AA). LDH: the GG genotype demonstrated significantly lower levels than both AG and AA genotypes (GG < AG, GG < AA). Alanine aminotransferase (ALT), aspartate aminotransferase (AST), creatinine (CRE), total cholesterol (TC), triglycerides (TG), high-density lipoprotein (HDL), and creatine phosphokinase (CPK): the GG genotype exhibited significantly higher levels than AG and AA genotypes, with GG > AG > AA.

## 4. Discussion

This study commenced with the screening and validation of miR-SNP loci. Using whole-genome chicken DNA as the template, primers were designed to amplify target fragments encompassing the *pre-miR-3528* sequence. Following purification and sequencing, a biallelic polymorphism (A/G) at nucleotide position +12 bp within the *gga-miR-3528* gene was identified. Subsequently, specific primers flanking this polymorphic site were designed for amplification. High-throughput genotyping of the *miR-3528* SNP locus (rs14098602 A/G) across the Gushi × Anka F_2_ resource population was performed using the MassArray system (employing MALDI-TOF MS coupled with single-base extension). Association analyses between this SNP and comprehensive phenotypic data from the F_2_ resource population (growth traits, body measurement traits, carcass traits, meat quality traits, and serum enzyme activities) yielded the following key results: ① Growth traits: a significant association was observed between the *gga-miR-3528* SNP and growth traits. The GG genotype was associated with significantly increased body weight at hatching, 2 weeks, and 4 weeks, as well as evisceration weight at 12 weeks (GG > AG > AA). ② Body measurement traits: individuals carrying the GG genotype consistently exhibited larger body dimensions compared to AG or AA genotypes across most measured traits. ③ Meat quality traits: subcutaneous fat rate, significantly reduced in GG individuals (GG < AG < AA). Pectoral muscle density and pectoral muscle area percentage: significantly elevated in the GG genotype. Leg muscle area percentage and body weight post-de-feathering: maximized in the GG genotype (GG > AG > AA). Pectoral muscle pH and leg muscle pH: minimized in the GG genotype (GG < AG < AA). Pancreas weight ratio: highest in the GG genotype (GG > AA, GG > AG). ④ Serum parameters: albumin, globulin, and cholinesterase: concentrations were significantly lower in the GG genotype compared to AG and AA genotypes (GG < AG < AA). Lactate dehydrogenase (LDH): activity was significantly lower in the GG genotype compared to both AG and AA genotypes (GG < AG, GG < AA). These comprehensive findings demonstrate significant associations between the rs14098602 SNP and critical performance traits in chickens, including hatching weight, body weight at 2 and 4 weeks, evisceration weight, subcutaneous fat rate, pectoral muscle density, serum total protein, serum albumin, serum globulin, serum cholinesterase, and serum lactate dehydrogenase.

Body weight serves as a critical selection criterion in chicken breeding programs. For example, SNP rs312619270 is a promising candidate molecular marker for body weight selection in chickens [18]. Therefore, its implementation in marker-assisted selection and as a molecular breeding marker for body weight traits in livestock and poultry holds significant potential for efficiently establishing genetically superior breeding populations [6,19]. The GG genotype demonstrates significant comprehensive trait advantages. In terms of growth and slaughter performance, compared to the AA genotype, GG individuals exhibited increased body weight at hatch (0 d) by 4.1% (*p *< 0.01) and at 2 weeks by 4.2% (*p *< 0.01), with eviscerated weight (EW) elevated by 2.9% (*p *< 0.05). Relative to the AG genotype, body weight gain from 0 to 4 weeks ranged from 3.9% to 7.5% (*p *< 0.05), highlighting superior early-growth performance. The weight-gain advantage range covers the core growth period. In terms of meat quality improvement, the GG genotype significantly reduced subcutaneous fat rate (SFR) by 31.4% (*p *< 0.05) and increased pectoral muscle density (PMD) by 15.2% (*p *< 0.05) compared to the AA genotype, achieving synergistic optimization of “fat reduction and lean muscle enhancement”. In terms of metabolic regulation, serum total protein (TP) decreased by 6.5–9.7% in GG individuals compared to AA/AG genotypes (*p *< 0.01), indicating accelerated protein turnover. Lactate dehydrogenase (LDH) activity decreased by 6.0% relative to the AG genotype (*p *< 0.05), reflecting improved energy metabolism efficiency. Although there was no significant difference in body size traits at 12 weeks of age, the GG genotype for shank length, shank girth, chest depth, body slanting length, and pelvis breadth was higher than the AA/AG genotype. This genotype overcomes the traditional trade-offs among growth rate, body fat deposition, and protein metabolism in breeding, providing a molecular marker foundation for developing ‘fast-growing, lean-meat, high-efficiency’ broilers.

SNPs refer to variations involving the substitution of a single nucleotide at specific positions within the genome. These variations exhibit a high degree of genetic diversity within populations. SNPs can affect the processing of miRNAs, thereby influencing the expression of target genes [4]. The discovery and application of SNPs have thus become indispensable tools for enhancing breeding efficiency and elucidating the genetic basis of biodiversity and complex traits. Within the specific domain of meat quality trait research, SNPs hold significant potential, particularly for improving meat product quality and production efficiency. While these findings highlight the potential of rs14098602 as a molecular marker, further validation in commercial purebred lines and diverse populations is essential for practical breeding applications.

MiRNAs play pivotal regulatory roles in muscle growth and development. Specifically, *miR-125a* functions as a suppressor of adipogenesis [20], inhibiting lipid deposition through the suppression of adipocyte differentiation. The *miR-206* and *miR-1* family members are key regulators of muscle development and meat quality formation in turkey (Meleagris gallopavo) [21]. In cattle, *bta-miR-2888* and *bta-miR-2309* have been identified as potential modulators of meat quality, where seed-region SNPs influence meat quality traits by altering miRNA-target gene interactions [22]. Research demonstrates that *miR-125a-5p* exerts critical control over porcine intramuscular fat (IMF) deposition and fatty acid composition by targeting the *KLF13* and *ELOVL6* genes, thereby influencing the proliferation and differentiation of intramuscular preadipocytes and fatty acid profiles, ultimately significantly impacting pork quality [20]. A multi-omics approach systematically analyzing associations between miRNA-related SNPs (miR-SNPs) and fatty acid composition in Nelore cattle muscle identified significant genetic variants and regulatory molecules—specifically, *bta-miR-2419-3p*, *bta-miR-193a-2*, and *bta-miR-1291*—associated with meat quality traits, providing novel insights into the genetic regulation of muscle fatty acid composition in cattle [23]. Furthermore, *miR-204* has been shown to play a significant role in muscle development, forming multiple circRNA-miRNA-mRNA regulatory axes [24]. The relationship between chicken traits and SNPs provides valuable breeding information for improving the production performance and economic benefits of poultry. The polymorphism of the *CD36* gene affects the carcass traits of chickens [25]. The expression regulation of the *ELOVL* gene has potential effects on the growth of poultry and the lipid deposition in different tissues. Genetic variations in *ELOVL3* can assist in marker-assisted selection for chicken meat quality [26]. The significance of the *LncEDCH1* gene and its related SNPs in chicken meat quality traits provides new molecular markers for precise poultry breeding [27]. Regarding the economic traits of poultry, especially the growth rate and meat quality of broilers, the application of miRNA SNP provides a new direction for breeding [28].

Serum biochemical indicators represent blood parameters intrinsically linked to physiological traits. Serum enzymes, responsible for catalyzing diverse physiological and biochemical processes within the animal body, exhibit levels and activities that reflect underlying growth and developmental and metabolic status. These parameters directly mirror animal physiological function, are crucial for normal growth and development, and are often regarded as a direct reflection of animal metabolism and health, thereby serving as significant diagnostic indicators. Traditional breeding methodologies, primarily reliant on phenotypic selection, possess inherent limitations. Traits such as fat composition, immunity, and others are challenging to measure directly, often requiring sib testing or slaughter assays, which result in prolonged generation intervals and high costs. Consequently, within the chicken breeding industry, there is a compelling need to develop convenient measurement parameters capable of reflecting physiological fat metabolism, immune function, and related processes. Serum biochemical indicators, recognized as a direct reflection of animal metabolic and health status, hold substantial significance in livestock and poultry production research. Blood collection is relatively straightforward, and the analysis of serum biochemical parameters facilitates indirect selection for specific traits, assessment of animal health, and early diagnosis of diseases, and effectively mitigates the drawbacks of traditional breeding—namely, its lengthy cycles and high costs [29].

Variations in serum enzyme activity—whether elevated or reduced—observed among different genotypes represent changes within the normal physiological range. At these levels, physiological function remains uncompromised across genotypes. Distinct chicken breeds, owing to their diverse genetic backgrounds, exhibit inherently different growth and metabolic characteristics, consequently manifesting varied serum biochemical profiles.

In this study, seventeen serum biochemical parameters were measured in chickens from the Gushi × Anka F_2_ resource population. Association analyses were performed between these parameters and the *gga-miR-3528* SNP genotypes to determine whether the SNP influences serum biochemistry. Our findings reveal that the GG genotype of the *gga-miR-3528* SNP significantly optimizes protein, lipid, and energy metabolism—likely through the modulation of key enzyme activities. This SNP thus represents a valuable molecular tool applicable to marker-assisted selection (MAS), precision nutritional regulation, optimization of health management protocols, and enhancement of meat quality and yield. The generalizability of these results beyond the Gushi × Anka F_2_ cross requires caution. Future studies should validate rs14098602 effects in standardized commercial breeds (e.g., Cobb, Ross) under intensive farming conditions. The rs14098602 (A > G) SNP in *gga-miR-3528* significantly enhances early growth, reduces subcutaneous fat, and optimizes serum enzymes in chickens. For industry: breeders should adopt GG genotype selection to improve lean-meat yield and growth efficiency. For research: future work must clarify the SNP’s mechanism (despite unaltered miRNA structure) and validate effects across breeds. This SNP provides a genetic tool for sustainable poultry production.

Leveraging this SNP as a biomarker for biochemical traits and a functional candidate gene offers a pathway to directly improve chicken growth rate, feed efficiency, and overall health status. This approach holds substantial promise for facilitating the advancement of efficient and sustainable intensive poultry-production systems. The low frequency of the GG genotype may limit the statistical power to detect smaller effects. While significant associations were observed for key traits, results for the GG group should be interpreted with caution and validated in larger, independent chicken populations.

Collectively, these results suggest that the rs14098602 polymorphism may modulate chicken growth and development, meat quality characteristics, and serum biochemical profiles. This strongly implicates the *gga-miR-3528* gene in playing a crucial regulatory role in chicken growth and developmental processes.

## 5. Conclusions

These findings indicate that this SNP plays a critical regulatory role in chicken growth and development. The *gga-miR-3528* gene may serve as a molecular marker for marker-assisted selection, providing an effective tool for enhancing economically valuable traits and facilitating commercial breeding programs.

## Figures and Tables

**Table 1:** Association of *gga-miR-3528* (+12 A > G) polymorphism with growth and carcass traits.

Traits	Mean ± SE			*p*-Value
	AA	AG	GG	
SEW (g)	1106.10 ± 182.75	1102.39 ± 188.64	1139.32 ± 149.89	0.429
EW (g)	924.69 ± 156.98 b	925.65 ± 163.75 b	951.32 ± 129.55 a	0.036 *
BMW (g)	70.64 ± 15.01	72.10 ± 16.69	74.13 ± 17.10	0.092
LMW(g)	98.00 ± 19.92	98.85 ± 19.80	102.88 ± 18.48	0.234
CW (g)	1218.79 ± 194.66	1212.37 ± 199.56	1259.62 ± 176.48	0.756
0BW (g)	30.85 ± 2.83 b	30.30 ± 2.76 b	32.12 ± 2.55 a	0.002 **
2BW (g)	123.63 ± 20.00 b	119.87 ± 17.90 b	128.85 ± 14.71 a	0.005 **
4BW (g)	324.60 ± 49.57 b	317.81 ± 45.15 b	330.07 ± 41.55 a	0.037 *
6BW (g)	567.06 ± 107.64	557.39 ± 107.19	572.21 ± 105.96	0.057
8BW (g)	819.49 ± 140.44	814.49 ± 141.48	820.88 ± 156.56	0.209
10BW (g)	1118.61 ± 182.14	1112.18 ± 180.41	1123.83 ± 165.40	0.214
12BW (g)	1357.20 ± 220.90	1344.31 ± 221.32	1383.76 ± 196.16	0.333

Note: SEW = semi-evisceration weight; EW = evisceration weight; BMW = breast muscle weight; LMW = leg muscle weight; CW = carcass weight. 0, 2, 4, 6, 8, 10, 12BW = body weight at the age of 0 day, 2, 4, 6, 8, 10 and 12 weeks, respectively. “a, b” indicate values within a row with no common superscript differing significantly (*p *< 0.05). ** *p *< 0.01, * *p* < 0.05.

**Table 2:** Association of *gga-miR-3528* (+12 A > G) polymorphism with body size traits.

Traits	SNP Genotype (Mean ± SE)			*p*-Value
	AA	AG	GG	
0SL (cm)	2.598 ± 0.018	2.575 ± 0.008	2.627 ± 0.026	0.578
4SL (cm)	5.502 ± 0.039	5.474 ± 0.059	5.604 ± 0.163	0.755
8SL (cm)	7.910 ± 0.044	7.920 ± 0.065	8.030 ± 0.136	0.834
12SL (cm)	9.359 ± 0.043	9.354 ± 0.059	9.495 ± 0.153	0.771
4SG (cm)	2.698 ± 0.010	2.674 ± 0.014	2.700 ± 0.050	0.393
8SG (cm)	3.418 ± 0.013	3.401 ± 0.019	3.459 ± 0.065	0.530
12SG (cm)	3.841 ± 0.015	3.828 ± 0.022	3.874 ± 0.066	0.752
4CD (cm)	4.840 ± 0.032	4.880 ± 0.045	4.730 ± 0.148	0.502
8CD (cm)	6.530 ± 0.044	6.520 ± 0.064	6.600 ± 0.185	0.931
12CD (cm)	7.862 ± 0.039	7.901 ± 0.051	8.022 ± 0.092	0.558
4CB (cm)	4.085 ± 0.024	4.080 ± 0.034	4.077 ± 0.065	0.989
8CB (cm)	5.671 ± 0.029	5.689 ± 0.037	5.741 ± 0.104	0.817
12CB (cm)	6.330 ± 0.031	6.330 ± 0.048	6.220 ± 0.132	0.732
4BBL (cm)	6.192 ± 0.025	6.233 ± 0.038	6.277 ± 0.094	0.524
8BBL (cm)	8.889 ± 0.036	8.956 ± 0.053	8.786 ± 0.209	0.433
12BBL (cm)	10.969 ± 0.038	11.037 ± 0.056	10.891 ± 0.154	0.504
4PA (°)	74.030 ± 0.245	74.180 ± 0.260	72.410 ± 0.818	0.224
8PA (°)	76.540 ± 0.256	76.360 ± 0.258	75.910 ± 0.767	0.780
12PA (°)	79.150 ± 0.197	79.180 ± 0.288	78.870 ± 0.803	0.942
4BSL (cm)	11.543 ± 0.161	11.385 ± 0.055	11.427 ± 0.150	0.786
8BSL (cm)	16.231 ± 0.059	16.214 ± 0.083	16.241 ± 0.229	0.984
12BSL (cm)	19.763 ± 0.064	19.704 ± 0.085	19.804 ± 0.238	0.845
4PB (cm)	5.180 ± 0.036	5.150 ± 0.034	5.270 ± 0.080	0.694
8PB (cm)	6.894 ± 0.043	6.862 ± 0.050	6.959 ± 0.165	0.830
12PB (cm)	8.690 ± 0.040	8.586 ± 0.062	8.750 ± 0.171	0.307

Note: 0, 4, 8, and 12 represent weeks. SL = shank length; SG = shank girth, CD = chest depth; CB = chest breadth; BBL = breast-bone length, PA = pectoral angle; BSL = body slanting length, PB = pelvis breadth.

**Table 3:** Association of *gga-miR-3528* (+12 A > G) polymorphism with meat quality traits.

Traits	SNP Genotype (Mean ± SE)			*p*-Value
	AA	AG	GG	
SFR	1.050 ± 0.067 a	0.780 ± 0.079 b	0.720 ± 0.283 b	0.037 *
PWR	0.252 ± 0.003	0.249 ± 0.004	0.257 ± 0.013	0.774
WAD	1181.512 ± 9.806	1182.997 ± 13.810	1214.227 ± 37.196	0.753
IFW	0.774 ± 0.019	0.718 ± 0.019	0.724 ± 0.074	0.163
SFT	0.484 ± 0.017	0.436 ± 0.011	0.438 ± 0.037	0.138
PMpH	6.134 ± 0.038	6.110 ± 0.015	6.037 ± 0.052	0.745
LMpH	6.814 ± 0.129	6.646 ± 0.011	6.588 ± 0.034	0.610
LMFR	0.892 ± 0.002	0.891 ± 0.002	0.891 ± 0.005	0.682
PMFR	0.917 ± 0.002	0.917 ± 0.001	0.906 ± 0.004	0.163
LMAP (%)	66.252 ± 0.502	66.649 ± 0.668	65.865 ± 1.787	0.872
PMAP (%)	66.205 ± 0.459 b	67.195 ± 0.570 ab	70.372 ± 1.997 a	0.058
LMFD	39.333 ± 2.692	37.189 ± 0.534	37.174 ± 1.448	0.847
PMFD	45.674 ± 2.067	43.849 ± 0.524	45.009 ± 1.843	0.821
LMD	922.155 ± 15.890	969.665 ± 24.867	931.169 ± 52.051	0.244
PMD	602.432 ± 8.422 b	625.318 ± 14.777 ab	693.943 ± 47.040 a	0.041 *
LMWLR	16.335 ± 0.212	16.545 ± 0.294	15.843 ± 1.036	0.706
PMWLR	24.186 ± 0.244	23.414 ± 0.372	23.655 ± 1.313	0.206

Note: SFR = subcutaneous fat rate; PWR = pancreas weight ratio; WAD = weight after de-feathering; IFW = intermuscular fat width; SFT = subcutaneous fat thickness; PMpH = pectoral muscle pH; LMpH = leg muscle pH; LMFR = leg muscle fullness ratio; PMFR = pectoral muscle fullness ratio; LMAP = leg muscle area percentage; PMAP = pectoral muscle area percentage; LMFD = leg muscle fiber diameter; PMFD = pectoral muscle fiber diameter; LMD = leg muscle density; PMD = pectoral muscle density; LMWLR = leg muscle water loss rate; PMWLR = pectoral muscle water loss rate. “a, b” indicate values within a row with no common superscript differing significantly (*p *< 0.05). * *p *< 0.05.

**Table 4:** Association of *gga-miR-3528* (+12 A > G) polymorphism with serum enzyme activities.

Traits	SNP Genotype (Mean ± SE)			*p*-Value
	AA	AG	GG	
ALT (U/L)	1.790 ± 0.113	2.060 ± 0.148	2.150 ± 0.472	0.323
AST (U/L)	282.900 ± 3.052	287.320 ± 4.659	303.780 ± 14.468	0.243
γ-GT (U/L)	15.310 ± 0.298	16.000 ± 0.380	15.610 ± 1.155	0.384
AKP (U/L)	713.180 ± 24.875	679.910 ± 32.463	810.230 ± 104.601	0.420
TP (g/L)	43.824 ± 0.410	42.344 ± 0.539	39.583 ± 1.229 a	0.008 **
ALB (g/L)	16.884 ± 0.116 a	16.541 ± 0.139 b	15.835 ± 0.314 b	0.024 *
GLOB (g/L)	27.066 ± 0.350 a	25.943 ± 0.454 b	23.748 ± 1.046 b	0.019 *
CHE (kU/L)	1.951 ± 0.027 a	1.825 ± 0.033 b	1.730 ± 0.072 b	0.005 **
CRE (μmol/L)	3.710 ± 0.329	3.840 ± 0.609	4.620 ± 1.876	0.814
GLU (mmol/L)	8.793 ± 0.176	8.690 ± 0.262	8.780 ± 0.782	0.945
TC (mmol/L)	3.138 ± 0.038	3.166 ± 0.050	3.342 ± 0.164	0.410
TG (mmol/L)	0.412 ± 0.005	0.416 ± 0.007	0.420 ± 0.014	0.887
HDL (mmol/L)	1.955 ± 0.021	2.010 ± 0.028	2.122 ± 0.092	0.070
LDL (mmol/L)	1.034 ± 0.021	1.001 ± 0.031	1.035 ± 0.080	0.676
CPK (U/L)	7297.89 ± 95.24	7352.29 ± 136.75	7868.57 ± 478.30	0.370
LDH (U/L)	2755.96 ± 25.12 a	2852.82 ± 32.98 b	2682.61 ± 85.03 a	0.042 *
AMY (U/L)	431.98 ± 10.49	423.40 ± 13.05	431.61 ± 46.42	0.885

Note: ALT = Alanine Aminotransferase; AST = Aspartate Aminotransferase; γ-GT = Gamma-Glutamyl. Transferase; AKP = Alkaline Phosphatase; TP = Total Protein; ALB = Albumin; GLOB = Globulin; CHE = Cholinesterase; CRE = Creatinine; GLU = Glucose; TC = Total Cholesterol; TG = Triglycerides; HDL = High-Density Lipoprotein; LDL = Low-Density Lipoprotein; CPK = Creatine Phosphokinase; LDH = Lactate Dehydrogenase; AMY = Amylase. “a, b” indicate values within a row with no common superscript differing significantly (*p *< 0.05). ** *p *< 0.01, * *p *< 0.05.

## Data Availability

The data presented in this study are available on request from the corresponding author.
